# Plasmid Composition, Antimicrobial Resistance and Virulence Genes Profiles of Ciprofloxacin- and Third-Generation Cephalosporin-Resistant Foodborne *Salmonella enterica* Isolates from Russia

**DOI:** 10.3390/microorganisms11020347

**Published:** 2023-01-30

**Authors:** Anna Egorova, Andrey Shelenkov, Konstantin Kuleshov, Nina Kulikova, Aleksey Chernyshkov, Igor Manzeniuk, Yulia Mikhaylova, Vasiliy Akimkin

**Affiliations:** Central Research Institute of Epidemiology, Novogireevskaya Str., 3a, 111123 Moscow, Russia

**Keywords:** *Salmonella*, antibiotic resistance, whole-genome sequencing, genomic epidemiology, mobile elements, cephalosporins resistance, ciprofloxacin resistance

## Abstract

*Salmonella enterica* is an important foodborne pathogen worldwide. Ciprofloxacin and extended-spectrum cephalosporins are the common first-line antimicrobial drugs for the treatment of salmonellosis, antimicrobial resistance genes for which are mostly transferred via plasmids. The goal of this work was to perform genomic analysis of plasmids from foodborne *S. enterica* isolates obtained in Russia based on whole-genome sequencing. In the current study, 11 multidrug-resistant samples isolated in 2021 from 8 regions of Russia were selected based on their resistance to ciprofloxacin and third-generation cephalosporins (CIP-3rd). Whole-genome short-read sequencing (WGS) was performed for all isolates; the samples belonged to five different sequence types (ST32, ST469, ST11, ST142, and ST548) which had different profiles of antimicrobial resistance (AMR) and virulence genes. We have performed additional long-read sequencing of four representative *S. enterica* isolates, which showed that they carried pESI-like megaplasmids of 202–280 kb length harboring extended-spectrum β-lactamase genes, fluoroquinolone, tetracycline, and aminoglycosides resistance genes, as well as several virulence determinants. We believe that the WGS data obtained will greatly facilitate further studies of foodborne *S. enterica* isolates epidemiology in terms of their self-transmissible plasmid composition that mediated antimicrobial resistance and virulence determinants conferring selective advantages of this important bacterial pathogen.

## 1. Introduction

The association between the consumption of particular food and human diseases was revealed by ancient Greeks more than 2000 years ago [1]. Foodborne pathogens, including viruses, bacteria, and eukaryotic parasites, can be ingested with food and usually multiply themselves within the human host during a period between several hours to several days, or even months until the symptoms occur. Each year, about 10% of people worldwide get ill from food contaminated with microbial or chemical agents, resulting in 600 million illnesses and 420,000 deaths globally [2]. Heretofore, more than 250 foodborne diseases have been revealed, and bacteria are the most common cause of them [3], making the surveillance of important bacterial pathogens essential for outbreak prevention.

Non-typhoidal *Salmonella enterica* is one of the most important global foodborne pathogens, which usually causes food poisoning and can infect various animals. Salmonellosis is the second most common gastrointestinal infection in humans after campylobacteriosis in the European Union [4] and is even a leading reported foodborne illness in some parts of the USA [5]. In the Russian Federation, salmonellosis remains an important cause of foodborne outbreaks, being third in the incidence among infections with a fecal–oral transmission mechanism [6].

The major sources of *S. enterica* foodborne contamination are poultry, meat, and their products [7,8]. Depending on the pathogenesis and degree of invasion *S. enterica* could lead to foodborne gastroenteritis, for the treatment of which antimicrobial drug therapy is needed to be prescribed. At the same time, the spreading of antimicrobial drug-resistant (AMR) strains remains a problem in the prevention and treatment of salmonellosis [9]. According to the Global Antimicrobial Resistance and Use Surveillance System (GLASS), the monitoring of extended-spectrum β-lactamases (ESBL), carbapenemases, colistin resistance, and fluoroquinolones resistance is required as a primary goal of epidemiological surveillance [10]. Fluoroquinolones are usually the drugs of choice for salmonellosis treatment in adults, whereas extended-spectrum cephalosporins (ESC) are used in children [11]. However, the application of these drugs becomes unsuitable as the ESC and quinolone resistance increases.

Extended-spectrum β-lactamases (ESBLs) represent the group of enzymes produced by Gram-negative bacteria which cause resistance to almost all known β-lactam antibiotics. The emergence of strains with ESC resistance has been reported worldwide [7,12,13]. Two major ESC resistance mechanisms have been identified in *Salmonella*. The first one is the expression of AmpC-like β-lactamases (TEM and SHV families) which hydrolyze the cephamycins, and the second is the production of ESBLs (CTX-M family) which can lead to oxyimino cephalosporins resistance [14].

The global spreading of ESBL genes is associated with the mechanism of horizontal transfer through plasmids. Moreover, the pathogenesis of microorganisms depends on multiple virulences and AMR genes mostly associated with plasmids [15]. A number of reports described the detection of megaplasmids harboring ESBL-encoding genes in *S. enterica* from Italy, Switzerland, and the USA [7,16,17]. Furthermore, recently pESI-like megaplasmids were revealed in Russia [18,19]. These plasmids are conjugative with up to 300 kb length and harbor AMR genes including ESBL genes, as well as genes conferring resistance to tetracyclines, sulphonamides, and aminoglycosides [20].

Apparently, resistance to β-lactams, especially ESC, and quinolones in *Salmonella* has become a significant public health concern worldwide [7,21]. Therefore, the present study aimed at an in-depth characterization of foodborne fluoroquinolone- and ESC-resistant *S. enterica* isolates from eight different regions of Russia and their plasmid replicons, which mediated antimicrobial resistance and virulence genes, using short-read (Illumina) and long-read (MinION) whole genome sequencing (WGS). We believe that the data on plasmid composition and their AMR and virulence gene content will facilitate better epidemiological surveillance of ESBL-producing *S. enterica* strains and further elucidate the antibiotic resistance mechanisms of this important pathogen.

## 2. Materials and Methods

### 2.1. Sample Collection, Species Identification, and Antibiotic Susceptibility Detection

A total of 255 *Salmonella* isolates were obtained from the samples collected by Russian Federal Centers of Hygiene and Epidemiology as a part of regular food monitoring activities in 2021. All the isolates were tested for susceptibility/resistance to the following drugs by VITEK 2 system (bioMerieux, Marcy-l’Étoile, France): ampicillin (AMP), ampicillin/sulbactam (SAM), cefepime (FEP), ceftazidime (CZD/CAZ), cefuroxime axetil (CXM), ceftriaxone (CRO), imipenem (IPM/M), ertapenem (ETP), amikacin (AMK), gentamicin (GMN), tobramycin (TOB), chloramphenicol (CHL), nitrofurantoin (NIT), trimethoprim/sulfamethoxazole (SXT), and ciprofloxacin (CIP). The isolates possessing resistance to CIP, CZD/CAZ, and CRO were selected for the current study. A total of 11 *Salmonella* isolates from poultry products (n = 8) and meat products (n = 3) were identified down to a species level using the VITEK MS system (bioMerieux, Marcy-l’Étoile, France). Detailed sources of isolation are given in Table S1. Samples were collected from 8 geographic regions of Russia. Six of the isolates studied were also tested for cefoxitin (FOX), cefuroxime (CFM), nalidixic acid (NAL), and tetracycline (TET) resistance, while five isolates were additionally tested for cefoperazone/sulbactam (CFP). The assessment of resistance to antimicrobial compounds included in this study was conducted according to the European Committee on Antimicrobial Susceptibility Testing (EUCAST) standards v.10.0 (http://www.eucast.org, accessed on 7 October 2021).

### 2.2. DNA Isolation and Whole Genome Sequencing

Genomic DNA for short-read WGS was isolated with Ribo-prep Kit (Amplisens, Moscow, Russia) according to manufacturer’s instructions. Quantity was evaluated by fluorimetry with Qubit 4.0 (Thermo Fisher Scientific, Waltham, AS, USA). DNA was used for paired-end library preparation with Nextera XT Kit (Illumina, San Diego, CA, USA) according to the manufacturer’s recommendations. Short-read WGS was performed on the Nextseq 2000 platform (Illumina, San Diego, CA, USA). The quality and quantity of libraries were confirmed by capillary gel electrophoresis (Agilent Bioanalyzer 2100, Agilent, Santa Clara, CA, USA) and Qubit 4.0 (Thermo Fisher Scientific, Waltham, AS, USA).

Long-read WGS for the further characterization of plasmid’s structure was performed for selected isolates. In this case, DNA was isolated from bacteria with Blood and Tissue Kit (Qiagen, Venlo, The Netherlands), and libraries were prepared with the Rapid Barcoding Sequencing kit SQK-RBK004 (Oxford Nanopore, Oxford, UK). Size selection and clean-up steps were performed using AMPure XP (Beckman Coulter, Brea, CA, USA). Quantification of libraries was performed by Qubit 4.0 (Thermo Fisher Scientific, Waltham, AS, USA). Sequencing of long reads was performed on MinION (Oxford Nanopore, Oxford, UK) R9 SpotON flow cell. Base-calling of the raw MinION data was made using Guppy base-calling software version 5.0.16 (Oxford Nanopore, Oxford, UK) with default parameters, and demultiplexing was performed using Guppy barcoding software version 5.0.16 (Oxford Nanopore, Oxford, UK).

### 2.3. Analysis of Sequencing Data and Genome Assembly

Short-read genome assemblies were obtained using SPAdes version 3.15.2 [22] with default parameters. Hybrid short- and long-read assemblies were performed using Unicycler version 0.4.9 (normal mode) [23]. Contigs having a length of less than 500 bp were removed.

Assembly quality estimation, organism checking, and initial annotation were performed using the custom pipeline described earlier [24,25]. Enterobase website (https://enterobase.warwick.ac.uk/species/index/senterica, accessed on 20 October 2022) was used for sequence type detection using multilocus sequence typing (MLST) scheme. *Salmonella enterica* MLST schemes include the fragments of the following seven loci: *aroC*, *dnaN*, *hemD*, *hisD*, *purE*, *sucA*, and *thrA*. We used the Resfinder 4.0 database with default parameters for antimicrobial gene identification (https://cge.cbs.dtu.dk/services/ResFinder/, accessed on 20 October 2022). AMRFinderPlus (version 3.11.2) [26] was used to predict point mutations. The presence of virulence factors was studied using the VFDB database (http://www.mgc.ac.cn/VFs/, accessed on 20 October 2022).

Plasmid sequences were revealed and typed using PlasmidFinder with default parameters (https://cge.cbs.dtu.dk/services/PlasmidFinder/, accessed on 20 October 2022). Plasmid visualization and comparison of plasmid composition with the pS7697-1 and pS7966-1 reference plasmids from NCBI was performed with BLAST Ring Image Generator (BRIG).

The assembled genome sequences for all isolates were uploaded to the NCBI Genbank under the project number PRJNA900218.

To build the phylogenetic tree representing the relations between the isolates based on core genome sequence, we used Roary version 3.13.0 (parameters: -i 95 -cd 99, no paralogs allowed in core genes) [27] and RAxML version 8.2.11 [28] software with default parameters (model used: -m GTRGAMMA).

Detection of cgMLST profiles was performed using MentaList (https://github.com/WGS-TB/MentaLiST, version 0.2.4, accessed on 12 December 2022, default parameters) [29] using the scheme obtained from cgmlst.org (https://www.cgmlst.org/ncs/schema/schema/4792159/, contained 3002 loci, last update 20 October 2022). The minimum spanning tree was built using PHYLOViz online (http://online.phyloviz.net, accessed on 15 December 2022) [30].

## 3. Results

### 3.1. Typing and Classification

**Figure 1:**
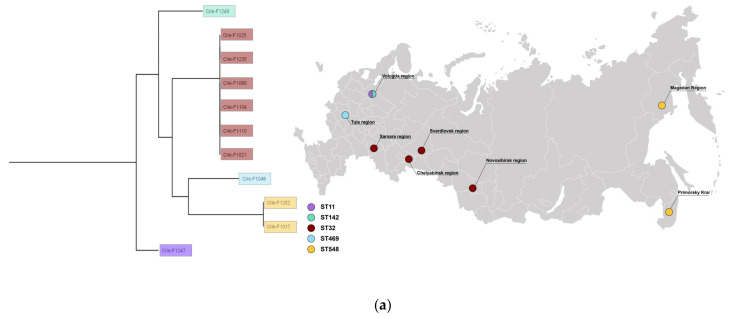

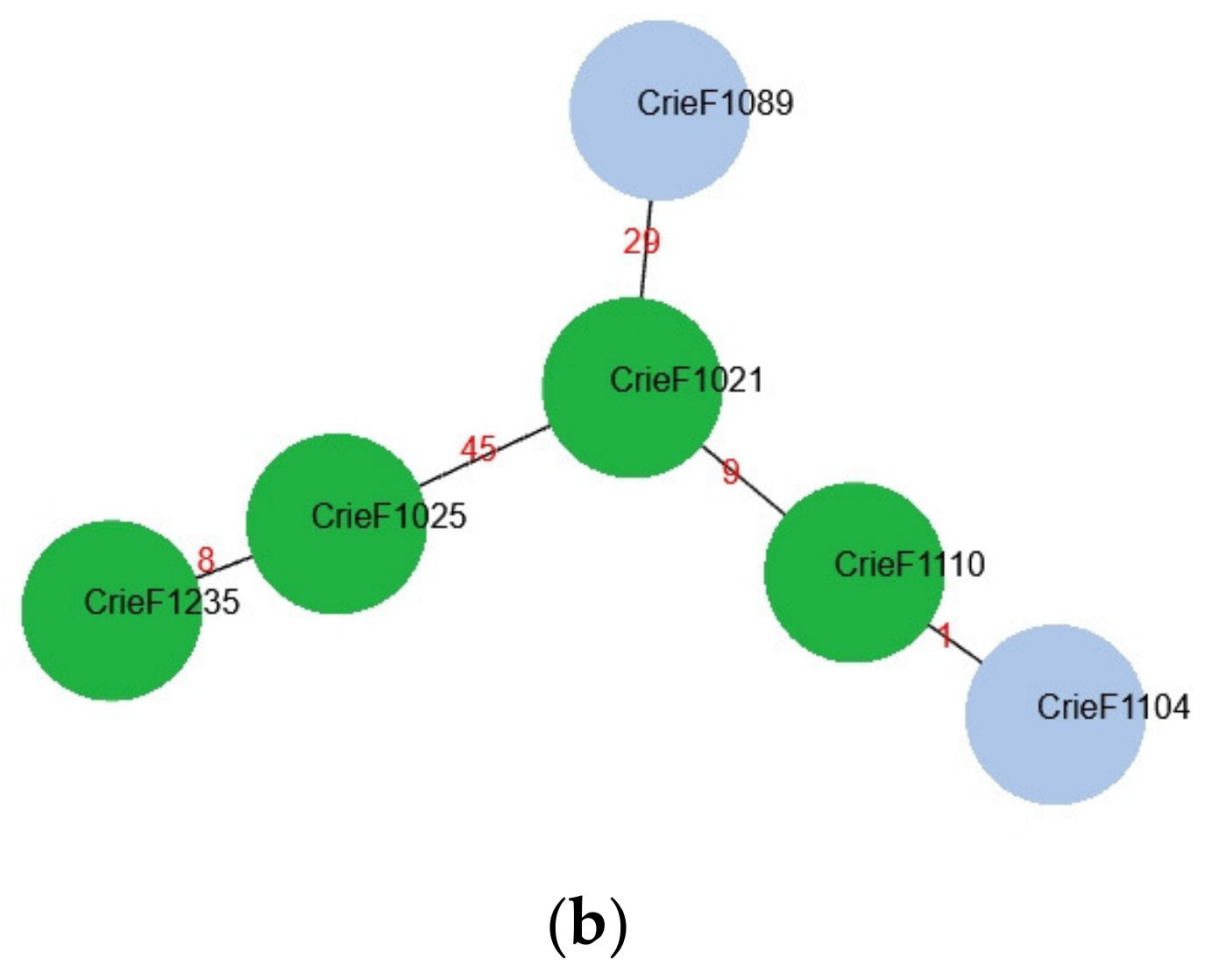
(**a**) Typing, geography, and phylogenetic analysis of CIP-3rd food *S. enterica* isolates studied. (**b**) A minimum spanning tree for CIP-3rd food *S. enterica* isolates with ST32. Red numbers indicate alleles discrepancies. The isolates for which the hybrid assemblies are available are shown in green.

The results of the isolate typing are presented in Table S1 and in Figure 1. The isolates belonged to five different sequence types: ST32 (n = 6), ST548 (n = 2), ST11 (n = 1), ST142 (n = 1), and ST469 (n = 1). In addition, there were four O-types: O7 (n = 7), O21 (n = 2), O8, and O9. The combined data above corresponds to Infantis, Minnesota, Enteritidis, Rissen, and Hindmarsh serotypes (Table S1). Four isolates were collected from three geographic regions of the European part of Russia comprising ST32, ST469, ST11, and ST142. Three geographic regions of the central part of Russia (Ural and Siberia) included only ST32. Interestingly, two isolates from the Magadan region and Primorsky krai had an identical ST548, which made us suggest *S. Minnesota* could spread in the far east of Russia.

In order to obtain additional information regarding bacteria similarity, a minimum spanning tree was built based on cgMLST profiles for six isolates of ST32 (Figure 1b). According to the phylogenetic analysis, a high similarity group of the same sequence type was revealed regardless of the different regions. The groups Crie-F1104—Crie-F1110—Crie-F1021, and Crie-F1025—Crie-F1235, respectively, could each belong to a single clone since they had a low number of allele differences.

### 3.2. Antibiotic Resistance Determination

**Figure 2:**
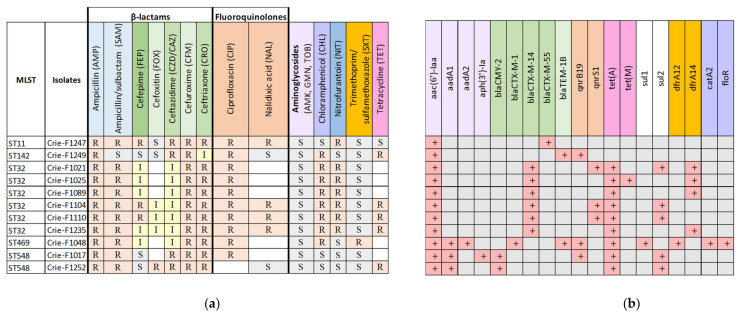
(**a**) Phenotypic and (**b**) genomic antimicrobial resistance profiles of foodborne *S. enterica* isolates resistant to third-generation cephalosporins. The same color indicates a particular antimicrobial group and the genes conferring resistance to the antimicrobials from this group. Light green indicates 2nd-generation cephalosporins, dark green—4th-generation cephalosporins, and CZD and CRO belong to 3rd-generation cephalosporins. White color shows missing results for particular antibiotics. R—Resistant, I—Intermediate, resistant with higher concentrations (according to EUCAST), S—susceptible.

Since the goal of this work was to perform a comprehensive analysis of CIP-3rd isolates of food origin, a total of 11 *S. enterica* isolates were tested for antibiotic susceptibility/resistance both by boundary concentration method using VITEK 2 system [31] and bioinformatics analysis to search for known acquired resistance genes in genomic sequences. The phenotypes and genotypes of the 11 isolates studied are presented in Figure 2.

The main selection criteria for the isolates studied were AMR to third- and/or fourth-generation cephalosporins. Thus, all 11 isolates were resistant to CZD, CRO, CFM (which belong to third- and second-generation cephalosporins), CIP and AMP, and only Crie-F1249 showed susceptibility to SAM. Additionally, the boundary concentration method identified resistance to FEP (fourth-generation cephalosporin), and CHL for eight isolates, and to NIT- for seven isolates (Figure 2a). Moreover, according to WGS data (Figure 2b), we revealed AMR determinants of such a resistance including β-lactamase genes. For example, all *S. Infantis* (ST32) isolates carried the *bla_CTX-M-14_* gene which was responsible for the third-generation cephalosporins resistance [32]. The isolates of ST548, ST469, and ST11 carried β-lactamase genes *bla_CMY-2_, bla_CTX-M-1_, bla_TEM-1B_*, and *bla_CTX-M-55_*, respectively. Since ESBL genes are located mostly on plasmids [33,34], further hybrid genome assemblies of representative isolates allowed us to obtain deeper insights into plasmid sequences and structure that could facilitate elucidating mechanisms of resistance acquisition by *S. enterica* isolates.

Furthermore, all FEP-resistant samples carried the members of the *bla_CTX-M_* gene family regardless of the sequence types of the isolates and geographic origin. Such dissemination raises concerns and requires further epidemiological surveillance of AMR spreading. Moreover, all the isolates under investigation were characterized by CIP resistance, however, the presence of AMR genes to fluoroquinolones (*qnrB19*) and quinolones (*qnrS1*) was revealed only in two and three isolates, correspondingly (Figure 2b). We revealed point mutations in a chromosomal gene *gyrA* encoding DNA gyrase subunit A which is involved in the most common mechanism of quinolone resistance in *Salmonella* [35] for all seven isolates not possessing AMR genes to quinolones (Table S2). Interestingly, ST142-isolate (Crie-F1249) possessed only *bla_TEM-1B_* despite the evident resistance to ESC (CRO and CZD/CAZ). We can suggest that the resistance to third-generation cephalosporins was associated with a strong p5-like promoter [36,37]. Therefore, the molecular mechanism of ESC resistance will possibly be clarified in further reports.

Nine out of eleven isolates studied contained tetracycline resistance genes. A combination of *dfrA12/dfrA14* genes with *sul1/sul2* confers resistance to trimethoprim-sulfamethoxazole. The isolate Crie-F1048 possessing *dfrA12/sul1* showed resistance to SXT, whereas Crie-F1021 with *dfrA14/sul2* did not show any phenotypic activity (Figure 2a). To summarize, most of the antimicrobial susceptibility profiles corresponded well to the AMR determinants revealed by WGS.

### 3.3. Virulence Genes

Virulence genes could be located on plasmids or within the chromosome and are involved in a complex of bacteria interactions with the host. The set of virulence determinants in the isolates studied was identical and extensive (up to 162) for the isolates of each ST. Table 1 presented genes that differ between sequence types and the full list of detected virulence genes is given in Table S3. Operons of adhesion, effector proteins of type III secretion system (*pipB*, *sspH2*) and type VI secretion system (*tae4*, *tssM*), and genes encoding membrane proteins were identified among them. The ST11 isolates were characterized by the most diverse virulence profile. Namely, the genes encoding invasion factors (*rck*), membrane unit *tssM*, *spv* gene cluster, which was encoded on a highly transmissible plasmid, and fimbrial protein *pefA-D* were identified. Samples with ST11 and ST142 additionally carried secreted effector *sseI* and superoxide dismutase *sodC1*. The isolates belonging to ST11, ST142, and ST469 carried the gene *entA* associated with ROX degradation, while only ST548 samples included the determinants encoding toxins (*cdtB, pltAB*). According to the hybrid assembly, the genes encoding yersiniabactin operon (*fyuA*, *irp12*, *ybtAEPQSTUX*) were of plasmid localization; the same picture was previously observed in *S. Infantis* isolates [19].

### 3.4. Plasmids

Plasmids are important vehicles in dissemination and acquisition of antibiotic resistance and virulence genes via horizontal gene transfer, and thus represent an important study object in the field of human healthcare [38]. The classification of plasmids has been determined by incompatibility patterns that refer to the inability of two plasmids to coexist stably over several generations in the same bacterial cell line [39]. Generally, closely related plasmids are incompatible by the presence of a replicon with the same specificity. In the current study, we identified 17 occurrences of 5 plasmid incompatibility groups for 11 CIP-3rd isolates listed in Table 2 below.

All CIP-3rd ST32 isolates were characterized by the presence of the only IncFIB plasmid replicon. At the same time, samples with ST548 carried IncC (Crie-F1017, Crie-F1252), ST469 isolate had several types of plasmid replicons simultaneously (Col(pHAD28) and IncI1), as well as ST11 (IncFIB, IncFII, IncI1) and ST142-isolates (Col(pHAD28), IncHI2, IncHI2A).

The combined use of short- and long-read sequencing allowed us to more accurately assemble the plasmid sequences of four representative isolates from our sample collection (Crie-F1017 and Crie-F1252 belonging to ST548, Crie-F1110 having the most abundant ST32, and ST142 isolate Crie-F1249). The in-depth genomic analysis confirmed that three CIP-3rd isolates carried megaplasmids with resistance and virulence gene patterns previously described as *S. enterica* plasmids (pS7697 for Crie-F1017 and Crie-F1252, and pS7966 for Crie-F1110), while the fourth annotated megaplasmid of Crie-F1249 aligned to *E. coli* plasmid pRHB41 related to IncHI2A group of incompatibility (Table S4). Their size ranged from 202 kb to approximately 280 kb (Table S4). According to the prediction by MOB-suite (Table 2), the big plasmid carried by Crie-F1110 belonged to the IncFIB group and was conjugative, as well as IncFIB for Crie-F1235, while other isolates (Crie-F1021 and Crie-F1025) possessed mobilizable plasmids with the same incompatibility group and some conjugative elements required for their mobility [40]. The sample Crie-F1017 carried conjugative pS7697-like megaplasmid of the IncC group, additional two plasmids of approximately 3 kb length previously found in *E. coli*, and an additional plasmid of a similar length as in Crie-F1252 was previously found in *Klebsiella pneumoniae*, while Crie-F1249 had a 3 kb length plasmid corresponding with *S. enterica* pF18S044 plasmid according to BLAST analysis against “nt” database. At the same time, the isolate Crie-F1110 possessed the only large plasmid similar to pS7966 harboring several AMR genes and virulence determinants.

**Figure 3:**
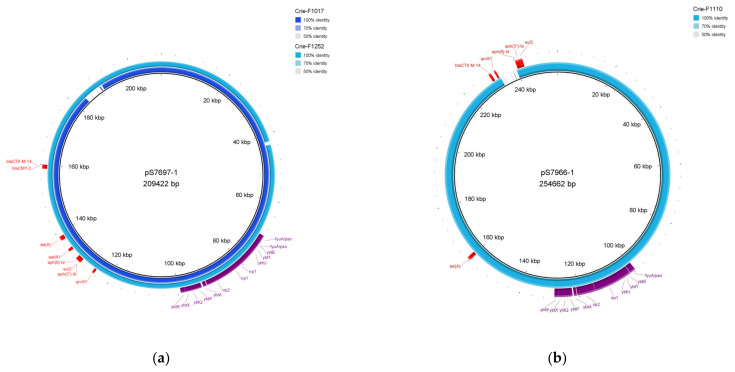
Megaplasmids determined by hybrid assembly. The plasmids were visualized via BRIG. (**a**) Plasmid of Crie-F1017 (locus identifier—NZ_JAPHVD010000004) and Crie-F1252 plasmid (locus identifier—NZ_JAPHUZ010000002) aligned to the pS7697-1 (209 kb) *S. enterica* plasmid. (**b**) Plasmid harbored by Crie-F1110 (NZ_JAPHVJ010000004) was aligned to pS7966-1 of *S. enterica*. Virulence genes located both on Crie-F1017 and Crie-F1252 plasmids are highlighted with violet color, AMR genes are highlighted with red.

Plasmids reconstructed in silico by the hybrid assembly are presented below in Figure 3. Visual modeling by the BRIG tool allowed us to reveal the acquisition of antimicrobial resistance determinants in the context of one sequence type. For instance, Crie-F1017 and Crie-F1252 (Figure 3a) correspond to the same plasmid pS7697 and obtained AMR genes to ESC (*bla_CMY-2_*), TET (*tetA*), SMX (*sul2*), and streptomycin (*ant(3″)-Ia*). Worth noting, reference plasmid pS7697 did not possess the *bla_CMY-2_* gene as opposed to ST548 isolates included in this report. This fact indicates that *bla_CMY-2_* is an acquired gene that possesses the ability to be horizontally transferred. Furthermore, Crie-F1017 carried additional AMR determinants to CIP (*qnrB19*) and kanamycin (*aph(3′)-Ia*), which is also indicated in the dissemination of genes found. Moreover, there were other references to megaplasmids of the isolates Crie-F1110 (Figure 3b) and Crie-F1249 (Figure S1) (pS7966 and pRHB41, correspondingly) with different lengths, AMR genes, and localization on a particular plasmid. The isolate of ST32 was characterized by the presence of six AMR determinants to TET, streptomycin, SMX, CIP, and ESC. Notably, *bla_CTX-M-14_* and *qnrS1* were not presented on the corresponding reference as described above for the isolates with ST548. Crie-F1249 obtained only *qnrB19* and *bla_TEM-1B_* AMR genes. According to the hybrid assembly, the genes encoding yersiniabactin operon (*fyuA*, *irp12*, *ybtAEPQSTUX*) were located on megaplasmids of the isolates Crie-F1017, Crie-F1252, and Crie-F1110 (Figure 3).

## 4. Discussion

In the current report, we analyzed eleven foodborne *S. enterica* isolates resistant to fluoroquinolones and third-generation cephalosporins obtained from various food products in eight different geographical regions of Russia as a part of epidemiological monitoring by the regional centers of Hygiene and Epidemiology. Isolates studied were clustered into five sequence types.

ST32 was the primary ST since it is prevalent in Russia [19], together with Europe and Japan [7,17,41,42]. Two isolates studied belonged to ST548, and other sequence types (ST11, ST469, and ST142) had single samples, while in China the predominant ST was ST11 (12.4%) and ST469 had 3.2% [21]. In silico MLST revealed the prevalent presence of ST11 (8/39; 20%) and ST 198 (8/39; 20%) in Tunisia, which were recovered mainly from poultry samples [35] and were also in concordance with other studies in Egypt [43]. Interestingly, a previous report from the European Union contained data regarding ESC-resistant *S. enterica* ST548 and ST15 imported with meat products from Brazil [44,45], while we received isolates with ST548 from the far east of Russia. However, there were reports from Brazil and the United Arab Emirates regarding MDR *S. enterica* ST548 with colistin resistance, which underlines the need for further monitoring in order to ensure food safety [46,47].

Since fluoroquinolones and beta-lactams are common first-line antimicrobial drugs for the treatment of salmonellosis [11], there is a worldwide problem with resistance to these antibiotics, its spreading, and studying the mechanisms of resistance. The *bla_CTX-M_* gene family determines a well-known mechanism of resistance to new types of cephalosporins [37]. However, almost twenty years ago, it was reported that ESBLs, which also inactivate ESC, were very rare in *Salmonella* genus [13]. Unfortunately, the current increase in *S. enterica* with ESBLs shows a dramatic dynamic. For instance, in Belgium and France, resistance to ceftriaxone and ceftiofur associated with *bla_CTX-M-2_* or *bla_CTX-M-9_* gene carried on large conjugative plasmids has been reported in *S. enterica* serovar Virchow isolates from poultry and humans [48,49]. *S.* Infantis isolates with third-generation cephalosporins resistance obtained from broilers in Japan carried *bla_CTX-M-14_* on 95-kb plasmids [50], and two *Salmonella* isolates from Brazil harbored *bla_CTX-M-2_* and *qnrB5* (fluoroquinolone AMR gene) associated with a 280 kb plasmid [51]. The data presented above corresponds with our determination that *bla_CTX-M_* from seven out of eleven foodborne *Salmonella* isolates were carried by large plasmids of 92-280 kb in length (Table 2). 

Resistance to ceftriaxone is most commonly mediated by a cephalomycinase (CMY) encoded by the *bla_CMY_* gene family [52]. The susceptibility test in the current report confirmed the resistance of all the isolates studied to CRO. However, only ST548 samples carried *bla_CMY-2_*. Thus, we can assume that CRO resistance was mediated by either *bla_CMY_* or *bla_CTX-M_* depending on a sequence type.

At the same time, among 40% of food-origin *bla_CMY-2_*-positive isolates from Brazil, 5% were of Heidelberg serotype and 35% were of Minnesota serotype [53]. Moreover, a global spread of *bla_CMY-2_*-encoded β-lactam resistance in *Salmonella* has been noted by the dissemination of *bla_CMY-2_* carried on conjugative plasmid [54]. In silico plasmid analysis in the current report showed that two isolates with *bla_CMY-2_* harbored megaplasmids of IncC incompatibility group carrying this gene. The same results were found in *E. coli* isolates from sheep which had the *bla_CMY_* gene and other AMR determinants located in an IncC plasmid [55]. Interestingly, there was a report that observed that *S. Heidelberg* carrying *bla_CMY-2_* was susceptible to carbapenem only [51]. Thus, epidemiological monitoring is required to control the dissemination of this determinant since it has predominantly plasmid localization and could lead to carbapenem resistance in the future. All of the above is consistent with previous reports showing that *bla_CTX-M_*-type and *bla_CMY-2_* are the most widely distributed ESBL-encoding genes in food-origin bacteria [37,51].

All but one (Crie-F1252) isolates studied showed phenotypic resistance to fluoroquinolones. Nevertheless, for six of them, WGS data confirmed this by the presence of corresponding AMR genes. AMRFinderPlus detected seven isolates with a point mutation in *gyrA*, as we previously suggested [19].

CHL was widely used in agriculture in the 1990s before it was found that CHL was cancerogenic [56], and it is still used in human medicine and in veterinary science [57,58]. Eight isolates out of eleven observed in the current report were resistant to this antimicrobial compound. We found that despite the resistance to CHL for eight isolates studied, only one of them included acquired genes responsible for this (*catA2* and *floR*), suggesting the presence of an alternative mechanism of chloramphenicol resistance. However, few reports were published concerning such a mechanism. For example, the number of CHL-resistant clinical isolates of *S. Typhimurium* from the UK producing chloramphenicol acetyltransferase was very low [59], as well as only 21.1% of the CHL-resistant foodborne isolates from Korea harbored corresponding AMR gene [60]. The alternative mechanism of resistance could be the lack of OmpF since it played a major role in the high level of chloramphenicol resistance in *S. Typhi* described by Toro et al. [61]. In addition, there was a suggestion that resistance to other antimicrobial drugs (polymyxin or β-lactams) could lead to cross-resistance to chloramphenicols [58,62,63,64].

We detected virulence genes related to *Salmonella* pathogenicity islands SPI-1 and SPI-2 in all 11 isolates observed. SPI-1 and SPI-2 encode a type three secretion system, effector proteins, and associated transcription factors that together enable host invasion [65]. Previously, we identified these virulence genes in *S. Infantis* [19], and they were shown to be the most common virulence determinants in this serotype [66].

No major differences between the virulence genetic factors were revealed in the 11 isolates studied, and the virulence patterns were quite conservative. Specific virulence gene pattern ESIv (Emergent *S. Infantis* virulence) [67] which consists of the genes encoding the virulent yersiniabactin operon (*fyuA, irp1, irp2, ybtAEPQSTUX*) and the genes associated with the fimbrial protein synthesis were identified in six isolates of ST32 and two of ST548. As previously reported [18], similar virulence gene patterns have been determined by the presence of a pESI-like megaplasmid. Interestingly, the yersiniabactin biosynthetic protein-coding cassette from *Klebsiella pneumoniae* was located in the chromosome, as mentioned earlier [68].

*Salmonella* plasmid virulence (*spv*) gene was found to be usual for *S. Enteritidis* since it occurred in all isolates studied by Andesfha et al. [69], and the isolate with ST11 (belonged to *S. Enteritidis*) in the current report harbored this gene as well. Moreover, a sample of ST11 studied had the *rck* motif, which encoded protein that consisted of several bacterial or phage outer membrane proteins involved in the virulence of Gram-negative pathogens [70]. Interestingly, it was determined that *S. Enteritidis* harbored the following genes: *sipA*, *sipD*, *sopD*, *sopB*, *sopE*, *sopE2*, *sitC*, *ssaR*, *sifA*, *spvC*, *pefA*, while *S. Infantis* had none of them [71]. Our data presented the same virulence profile for all genes described above regardless of the isolate ST.

The types of plasmid replicons determined in silico by us (IncI1, IncHI2A, IncHI2 IncC, IncFII, and IncFIB) have been reported quite frequently among Enterobacteriaceae [68,72] and play a crucial role in the spreading of integrons and resistance genes [73]. In addition, they are considered “epidemic resistance plasmids” that have been detected worldwide in Enterobacteriaceae of different origins and sources [73].

Megaplasmids analyzed in the current study were characterized by different profiles of AMR and virulence genes for each sequence type observed. According to hybrid assemblies of whole genomes for four representative samples, the isolate Crie-F1249 carried two plasmids (3 Kb and 223 Kb length), and an IncHI2A replicon (Table 2). It should be noted that IncHI plasmids are generally conjugative and large, containing up to 300 kb [74], which was confirmed by the data obtained by us. IncHI2 plasmids have a well-conserved backbone structure with regions of variation [75], and often carried *cat, strAB, tetAR, sul2, bla_TEM-1_* [34]. Recently, such plasmids were found to harbor ESBL and *qnr* genes [76]. The plasmids with a replicon type IncHI2 aligned with *S. enterica* megaplasmid pSal016 of 259 kb length possessed various AMR genes including *aadA22*, *aac(3)-IId*, *aph(3′)-Ia*, *bla_TEM–1B_*, *bla_CTX–M–55_*, *qnrS1* and other [33], while megaplasmid with replicon IncHI2A in our research carried *bla_TEM–1B_* and *qnrB19* genes only.

ST548-isolates (Crie-F1017 and Crie-F1252) contained conjugative plasmids with IncC replicon of 202-207 kb length, which is one of the most well-studied incompatibility groups found in *Salmonella* [77]. Megaplasmid observed in the current report carried such AMR genes as *ant(3″)-Ia*, *sul2*, *bla_CMY_*, and *tetA*. It was noted that these AMR genes are the most common for IncC [77]. IncC plasmids are large, low-copied, and frequently contain AMR genes [78].

The isolate Crie-F1110 harbored a plasmid of IncFIB replicon type which carried *aph(3”)-lb*, *sul2*, *tetA*, *qnrS1*, and *bla_CTX-M-14_*. Large conjugative megaplasmid pESI (plasmid for emerging *S. Infantis*) provides multidrug-resistance associated with *aadA1, bla_CTX-M-14_, dfrA14, sul1, tetAR* genes [18] which is in line with our data. Usually, pESI-like megaplasmids contain 2-5 AMR gene groups. The plasmids belonging to the incompatibility group IncFIB are able to encode both virulence factors and antimicrobial resistance genes [79], which is problematic if antimicrobial therapy is required.

This study reports the snapshot of ESC resistance dissemination in *S. enterica*. However, future retrospective studies from different sources are needed to assess the prevalence of plasmid-mediated ESC and ciprofloxacin resistance in *Salmonella* from Russia.

## 5. Conclusions

The current report presents the plasmid characterization of 11 foodborne fluoroquinolones and extended-spectrum cephalosporin (ESC)-resistant *S. enterica* isolates performed by short-read and long-read WGS. The isolates studied were obtained from various geographic regions of Russia. The isolates were characterized by their phenotypic and genomic AMR profiles, the presence of virulence factors, and plasmid properties including replicon sequences and in silico mobility prediction.

All of the 11 *S. enterica* isolates from food contained large plasmids with a length up to approximately 280 Kb which carried the ESC resistance genes belonging to *bla_CTX-M_* or *bla_CMY_* families. AMR genes for quinolones were also located on megaplasmids. However, not all AMR mechanisms were clarified (e.g., resistance to CHL and the occurrence of *catA* or *floR* did not match), and thus further studies will be required to establish the actual molecular mechanisms of resistance to therapeutic antimicrobial drugs and their dissemination. 

The data obtained will facilitate further studies in the epidemiology of *S. enterica* isolates, especially in the surveillance of various types of plasmids, their circulation in bacterial populations, and their characteristics in terms of AMR genes, virulence, and other pathogenic factor composition.

## Figures and Tables

**Table 1:** Virulence profiles of foodborne *S. enterica* isolates studied.

MLST	Isolates	Total Number of Genes Found	Virulence Genes																					
			allB	cdtB	entA	fepC	fyuA/psn; irp1,2; ybt	lpfA-E	mig-5	pefA-D	pipB2	pltAB	ratB	rck	shdA	sodCI	spvBCD	sseI/srfH	sseK1	sseK2	sspH2	tae4	tlde1	tssM
**ST11**	**Crie-F1247**	140	+	−	+	−	−	+	+	+	+	−	+	+	−	+	+	+	+	−	+	−	+	+
**ST142**	**Crie-F1249**	130	+	−	+	+	−	+	−	−	+	−	+	−	−	+	−	+	+	+	+	−	−	−
**ST32**	**Crie-F1021**	162	+	−	−	+	+	+	−	−	+	−	+	−	+	−	−	−	+	+	+	+	+	−
**ST32**	**Crie-F1025**	162	+	−	−	+	+	+	−	−	+	−	+	−	+	−	−	−	+	+	+	+	+	−
**ST32**	**Crie-F1089**	162	+	−	−	+	+	+	−	−	+	−	+	−	+	−	−	−	+	+	+	+	+	−
**ST32**	**Crie-F1104**	162	+	−	−	+	+	+	−	−	+	−	+	−	+	−	−	−	+	+	+	+	+	−
**ST32**	**Crie-F1110**	162	+	−	−	+	+	+	−	−	+	−	+	−	+	−	−	−	+	+	+	+	+	−
**ST32**	**Crie-F1235**	162	+	−	−	+	+	+	−	−	+	−	+	−	+	−	−	−	+	+	+	+	+	−
**ST469**	**Crie-F1048**	149	+	−	+	+	−	+	−	−	+	−	−	−	−	−	−	−	+	+	−	+	+	−
**ST548**	**Crie-F1017**	156	−	+	−	+	+	−	−	−	−	+	+	−	−	−	−	−	−	+	−	+	+	−
**ST548**	**Crie-F1252**	156	−	+	−	+	+	−	−	−	−	+	+	−	−	−	−	−	−	+	−	+	+	−

Genes highlighted in bold were located on megaplasmids according to hybrid assembly.

**Table 2:** Plasmid replicon sequences found in the isolates.

MLST	Isolates	Plasmid Replicons				Predicted Mobility
		IncF	IncC	Col	Others	
ST11	Crie-F1247	IncFIB, IncFII	−	−	IncI1-I	
ST142	Crie-F1249 *	−	−	Col(pHAD28)	IncHI2, IncHI2A	conjugative
ST32	Crie-F1021	IncFIB	−	−	−	mobilizable
ST32	Crie-F1025	IncFIB	−	−	−	mobilizable
ST32	Crie-F1089	IncFIB	−	−	−	non-mobilizable
ST32	Crie-F1104	IncFIB	−	−	−	
ST32	Crie-F1110 *	IncFIB	−	−	−	conjugative
ST32	Crie-F1235	IncFIB	−	−	−	conjugative
ST469	Crie-F1048	−	−	Col(pHAD28)	IncI1-I	non-mobilizable
ST548	Crie-F1017 *	−	IncC	Col(pHAD28)	−	conjugative
ST548	Crie-F1252 *	−	IncC	−	−	conjugative

The green and pink colors indicate correspondence for replicon type and prediction of mobility by MOB-suite; * indicates representative *S. enterica* isolates of food origin used for hybrid assembly.

## Data Availability

The assembled genome sequences for all isolates were uploaded to the NCBI Genbank, the project number PRJNA900218.

## Supplementary Materials

The following supporting information can be downloaded at: https://www.mdpi.com/article/10.3390/microorganisms11020347/s1, Figure S1: Plasmid of Crie-F1249 (locus identifier—NZ_JAPHVA010000002) aligned to the pRHB41 (223 kb) *E. coli* plasmid. The plasmid sequence was determined by hybrid assembly and was visualized via BRIG. AMR genes are highlighted with red; Table S1: The metadata and molecular typing for the foodborne *S. enterica* isolates studied; Table S2: Results of point mutation prediction by AMRFinderPlus; Table S3: Full data for virulence genes detected using the VFDB database in the foodborne *S. enterica* isolates; Table S4: Plasmids determined by hybrid assembly with AMR and virulence genes shown for four representative *S. enterica* isolates.
