# Supplementary material for: Plasmid Composition, Antimicrobial Resistance and Virulence Genes Profiles of Ciprofloxacin- and Third-Generation Cephalosporin-Resistant Foodborne Salmonella enterica Isolates from Russia
Source: Microorganisms. 2023 Jan 30;11(2):347. doi: 10.3390/microorganisms11020347 (PMC9961839; doi:10.3390/microorganisms11020347)

Supplementary Information for

Plasmid composition, antimicrobial resistance and virulence genes profiles of ciprofloxacin- and third-generation cephalosporin-resistant foodborne *Salmonella enterica* isolates from Russia

Anna Egorova *, Andrey Shelenkov, Konstantin Kuleshov, Nina Kulikova, Aleksey Chernyshkov, Igor Manzeniuk, Yulia Mikhaylova and Vasiliy Akimkin

Central Research Institute of Epidemiology, Novogireevskaya str., 3a, 111123 Moscow, Russia

* Correspondence: bioanna1995@gmail.com

Figure S1. Plasmid of Crie-F1249 (locus identifier - NZ_JAPHVA010000002) aligned to the pRHB41 (223 kb) *E. coli* plasmid. The plasmid determined by hybrid assembly and was visualized via BRIG. AMR genes are highlighted with red.

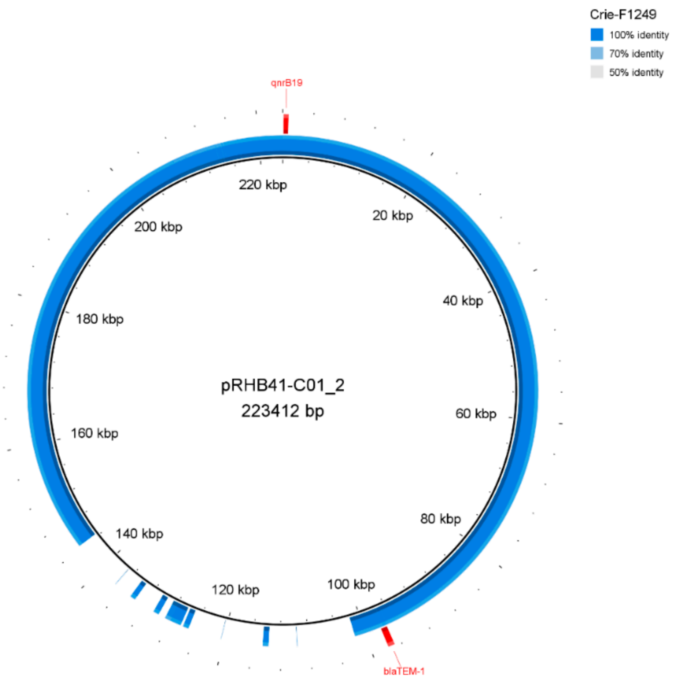

Supplement: Supplementary file 1 [file microorganisms-11-00347-s001.zip › Supplementary Information.pdf]
