# Supplementary figures and images for: Plasmid Composition, Antimicrobial Resistance and Virulence Genes Profiles of Ciprofloxacin- and Third-Generation Cephalosporin-Resistant Foodborne Salmonella enterica Isolates from Russia
Source: Microorganisms. 2023 Jan 30;11(2):347. doi: 10.3390/microorganisms11020347 (PMC9961839; doi:10.3390/microorganisms11020347)

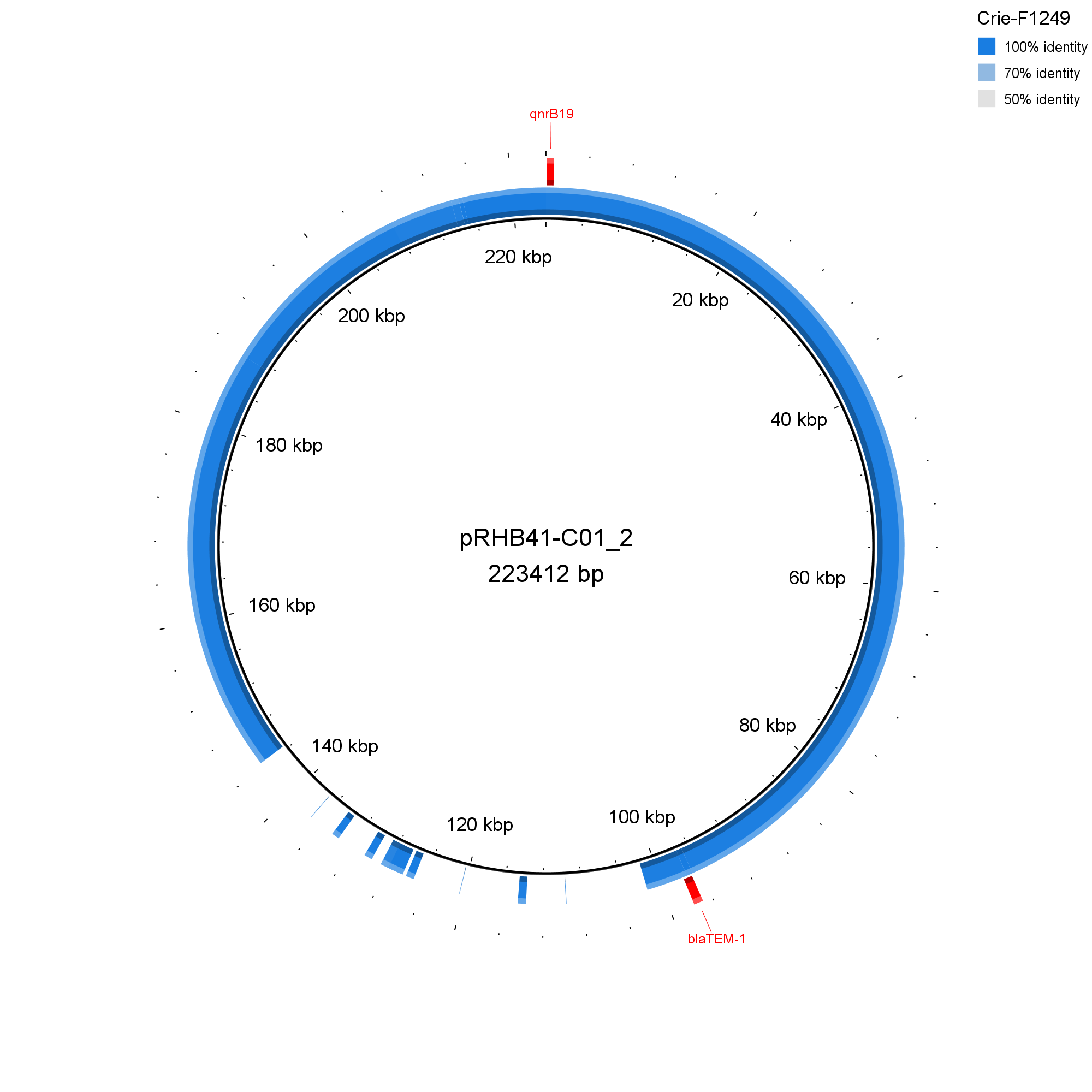

Supplement: Supplementary file 1 [file microorganisms-11-00347-s001.zip › Figure S1.png]
